# Prophylactic retrorectus mesh *versus* no mesh in midline emergency laparotomy closure for prevention of incisional hernia (PREEMER): study protocol for a multicentre, double-blinded, randomized controlled trial

**DOI:** 10.1093/bjsopen/zrab142

**Published:** 2022-01-28

**Authors:** Elisa Mäkäräinen, Matti Tolonen, Ville Sallinen, Panu Mentula, Ari Leppäniemi, Mirella Ahonen-Siirtola, Juha Saarnio, Pasi Ohtonen, Filip Muysoms, Tero Rautio

**Affiliations:** Department of Surgery, Medical Research Center, Oulu University Hospital, Oulu, Finland; Department of Surgery, Helsinki University Hospital, Helsinki, Finland; Department of Surgery, Helsinki University Hospital, Helsinki, Finland; Department of Surgery, Helsinki University Hospital, Helsinki, Finland; Department of Surgery, Helsinki University Hospital, Helsinki, Finland; Department of Surgery, Medical Research Center, Oulu University Hospital, Oulu, Finland; Department of Surgery, Medical Research Center, Oulu University Hospital, Oulu, Finland; Department of Surgery, Medical Research Center, Oulu University Hospital, Oulu, Finland; Hospital AZ Maria Middelares, Ghent, Belgium; Department of Surgery, Medical Research Center, Oulu University Hospital, Oulu, Finland

## Abstract

**Background:**

Despite the fact that emergency midline laparotomy is a risk factor for an incisional hernia, active research on hernia prevention in emergency settings is lacking. Different kinds of meshes and mesh positions have been studied in elective abdominal surgery, but no randomized controlled trials in emergency settings have been published thus far.

**Method:**

The PREEMER trial (registration number NCT04311788) is a multicentre, patient- and assessor-blinded, randomized controlled trial to be conducted in six hospitals in Finland. A total of 244 patients will be randomized at a 1 : 1 ratio to either the retrorectus mesh group, featuring a self-gripping prophylactic mesh, or to the no mesh (control) group, both closed by small-stitch 4 : 1 closure with continuous slowly absorbable monofilament suturing. The primary outcome of the PREEMER trial is the incidence of incisional hernia 2 years after surgery, which will be detected clinically and/or radiologically. Secondary outcomes are the Comprehensive Complication Index score, incidence of surgical-site infections and fascial dehiscence within 30 days of surgery; the incisional hernia repair rate and mesh- or hernia-related reoperations within the 2- and 5-year follow-ups; the incidence of incisional hernia within the 5-year follow-up; and quality of life measured by RAND-36, the Activities Assessment Scale and the PROMIS questionnaire within 30 days and 2 and 5 years from surgery. Additionally, health–economic explorative measures will be explored.

**Conclusion:**

The PREEMER trial will provide level 1 evidence on incisional hernia prevention in an emergency setting.

**Registration number:**

NCT04311788 (http://www.clinicaltrials.gov). Registered 7 March 2020.

## Teaser

The PREEMER trial is a multicentre, patient- and assessor-blinded, randomized controlled trial to be conducted in six hospitals in Finland. The trial will provide level 1 evidence on incisional hernia prevention in an emergency setting.

## Introduction

Emergency midline laparotomy is, in itself, a known risk factor of incisional hernia development with incidence of incisional hernia of up to 33 per cent^1–4^. However, no evidence-based recommendations have been given on the optimal technique for closing emergency midline laparotomy incisions^5,6^. A small-bite technique with a suture to wound length (SL : WL) ratio of at least 4 : 1 and a slowly absorbable monofilament suture is the current recommended technique for fascial closure in non-emergency settings^5^. The same method can also be utilized to close an emergency midline laparotomy to avoid an incisional hernia and fascial dehiscence^4,7^.

Prophylactic mesh augmentation in a non-emergency midline laparotomy appears both effective and safe in incisional hernia prevention^8^. Additionally, the emerging evidence suggests that synthetic meshes are safe in both contaminated and emergency surgery^9,10^. On the contrary, the current evidence does not support the use of biological meshes^11^.

There have, however, been only a few studies on incisional hernia prophylaxis within an emergency setting. In a recent systematic review and meta-analysis, the results of two studies, totalling 299 patients, were eligible to be analysed^2^. A case–control study from Switzerland reported an incisional hernia rate of 3.2 per cent (2 of 63 patients) in its intra-abdominal mesh group and 28.6 per cent (20 of 70 patients) in its sutured control group after emergency midline laparotomy for peritonitis^12^. A Spanish group exhibited similar results in their retrospective cohort study including patients with emergency midline laparotomies: an incisional hernia rate of 5.9 per cent (3 of 50 patients) in the onlay mesh group and 33.3 per cent (33 of 100 patients) in the control group^13^. There was no statistically significant difference in the incidence of surgical-site infection (SSI) or other complications when the prophylactic-mesh group was compared with the standard-closure group.

As an emergency laparotomy is a significant risk factor for incisional hernia, a mesh-augmented closure should be considered. Therefore, a randomized controlled trial (RCT) has been designed comparing prophylactic mesh with the best standard suturing technique within this challenging setting.

The objective of this study is to evaluate whether the rectorectus placement of a self-gripping polypropylene mesh (Progrip™; Medtronic) is safe and prevents an incisional hernia after emergency midline laparotomy. The results of mesh-augmented closure are compared with controls operated with no mesh by using the best standard 4 : 1 small-stitch suturing technique. The self-gripping mesh was chosen due to its indication for hernia prevention and easily standardized application. Onlay mesh has been associated with an increased risk of seroma^14^. As an emergency laparotomy, especially one at a contaminated surgical site, is prone to infections and seromas^2^, a retrorectus position was chosen for this study. This position also enables the skin to be left open in the cases of contamination level IV (dirty/contaminated).

## Method

This study is a multicentre, patient- and assessor-blinded, randomized controlled superiority trial conducted in Oulu, Helsinki, Tampere, and Turku university hospitals as well as Päijät-Häme and Seinäjoki non-university hospitals in Finland.

The coordinating centre for the trial is Oulu University Hospital. The composition of the coordinating centre consists of Chief of Surgery, Professor of Gastrointestinal Surgery, two consultants, biostatistician and research nurse. The first author and research nurse are responsible for day-to-day support for the trial. All other centres are advised to contact the first author (E.M.) for organizational support. As the trial is free of industrial sponsorship and no professional steering committees exist in Finnish hospitals, no commercial steering committee is nominated for the trial.

### Eligibility criteria

Participating investigators are qualified surgeons experienced in the surgical management of patients with emergency midline laparotomy and centres have a patient population large enough for the study requirements. All surgeons considered for participation must be experienced in closing the abdomen by 4 : 1 small-stitch technique and prophylactic self-gripping polyester mesh (Progrip™) placement. A detailed brochure with step-by-step pictures of midline laparotomy closure and mesh application is delivered to each participating hospital. The principal investigator may provide advice regarding mesh-application technique if desired.

The inclusion criterion is midline emergency laparotomy for any abdominal indication. Conversion from laparoscopy to laparotomy is accepted.

Exclusion criteria are previous ventral hernia repair with mesh in the midline, previous inguinal or femoral hernia repair using any technique with mesh is accepted; WHO class of physical activity 3–4 (rest time greater than 50 per cent of day in bed); relaparotomy within 30 days of previous abdominal surgery; indication for laparotomy is hernia-related; pregnant or suspected pregnancy; patient less than 18 years old; metastatic malignancy of any origin, but emergency operation with curative intent for intra-abdominal malignancy is accepted for inclusion; patients living geographically distant and/or unwilling to return for follow-ups; no informed consent provided; patient participates in other surgical RCT; planned or existing ostomy.

Intraoperative exclusion criteria applicable for both randomized groups are where the abdomen is left open; second-look laparotomy is planned; the mesh cannot be placed outside the intra-abdominal cavity or the anterior fascia cannot be closed; intra-abdominal non-curable malignancy is diagnosed during the operation; midline hernia is greater than 2 cm wide.

Patients fulfilling the inclusion criteria and not meeting the exclusion criteria will be offered the opportunity to participate by investigator or the surgeon on call when a decision for an emergency laparotomy is made.

### Interventions

In the control group, the fascia is closed using slowly absorbable monofilament suture with 4 : 1 small-stitch technique. In the intervention group, self-gripping mesh (Progrip™) in the rectorectus position was chosen to avoid need for separate attachment method of the mesh and to diminish the risk of seromas associated with onlay mesh^10^. The posterior layer of the rectus sheath is opened as close to the midline as possible without interrupting the midline. The space behind the rectus muscle is created mainly using a blunt dissection. Opening of the retrorectus space is achieved both cranially and caudally over the ends of the wound, if applicable. The posterior layer is closed using USP 0 or 2-0 slowly absorbable monofilament 4:1 small-stitch technique. The length of the wound is measured as well as the length of the suture material used. After ensuring that there will be no contact with the mesh and abdominal cavity, an 8 cm-wide self-gripping mesh (Progrip™) is applied on the posterior layer of the rectus sheath, extending over the opening at each end. The anterior layer of the rectus sheath is closed using slowly absorbable monofilament 2/0 or 0 sutures via the 4 : 1 small-stitch technique. The subcutaneous layer may be left temporarily open with vacuum-assisted closure or another wound dressing according to surgeons’ preference in contamination level IV. In contamination levels I–III (clean I, clean/contaminated II, contaminated III), the skin is closed according to the surgeons’ preference. Catalogues with operating pictures of the technique will be sent to all participating surgeons to standardize the procedure.

### Outcomes

#### Primary outcomes

The primary endpoint of this study is the incidence of incisional hernia, either symptomatic or asymptomatic, detected clinically and/or radiologically within 2 years after surgery and compared between the groups.

The definition and classification of an incisional hernia provided by the European Hernia Society (EHS) will be used to classify the primary outcome: ‘Any abdominal wall gap with or without a bulge in the area of a postoperative scar perceptible or palpable by clinical examination or imaging’^15^. In the case of inconsistencies between the clinical and radiological evaluations, or either clinical evaluation or imaging is missing for any reason, the following definitions of the primary endpoint will be used: if there is inconsistency between the ultrasound and CT scans, the result of the CT scan will be applied. If there is a suspicion of hernia based on clinical evaluation, but the ultrasound scan is negative, CT scan may be performed according to standard care of a patient (Table 1).

**Table 1:** Definition of primary endpoint

Clinical examination result	Imaging result	Primary endpoint
**Hernia**	Hernia	Hernia
**No hernia**	Hernia	Hernia
**Hernia**	No hernia	No hernia
**No hernia**	No hernia	No hernia
**Hernia**	Missing	Hernia
**No hernia**	Missing	No hernia
**Missing**	Hernia	Hernia
**Missing**	No hernia	No hernia

#### Secondary outcomes

The mean/median depending on the normality of the secondary outcomes is to be compared between the groups for Comprehensive Complication Index (CCI)^16^ and health–economic measures. The rates between the groups will be compared for the rest of the outcomes. The secondary outcomes are defined as: CCI within 30 days from surgery; surgical site infection (SSI) rate defined via the Centers for the Disease Control and Prevention (CDC) classification of SSI within 30 days of follow-up; fascial dehiscence defined as separation of facial closure within 30 days from surgery; incidence of incisional hernia defined according to definition by EHS within 5 years of follow-up; incisional hernia repair rate within 2 and 5 years from surgery; reoperations due to mesh or hernia within 2 and 5 years from surgery; quality of life (QOL): RAND-36, Activities Assessment Scale (AAS) and PROMIS) at 30 days and 2 and 5 years from surgery, compared between the randomization groups; health–economic explorative measures by cost–benefit analysis within the follow-up period for amount of time to create the retrorectal space and insert the mesh, duration of stay, costs of materials used to close the abdomen, duration of sick leave of a patient (retired and stay-at-home patients are excluded due to inability to estimate the length of sick leave), direct costs of hospital treatment caused by recurrence and reoperation.

The QOL questionnaire will be in official languages in Finland. AAS and PROMIS questionnaires were selected to measure the level of activity and functional outcome, although they are not validated in the desired languages. The results of QOL questionnaires will be compared between the randomization groups.

All related costs will be explored. The direct costs, such as the mesh, resources and hospital stay costs, are monitored, and the indirect costs from productivity losses of a patient are estimated.

The following costs of treatment for both groups will be analysed in detail: mesh and other materials used to close the abdomen; need for further surgery and medical treatment (all complications related to primary surgery, mesh-related need for surgery or other treatment, hernia-related need for surgery or any help from the medical system, duration of sick leave from work, need for rehabilitation before returning home, duration of stay at hospital).

### Participant timeline (*Table 2*)

The following data will be recorded prospectively using specific electronic case-report forms (eCRFs).

**Table 2:** Participant timeline

Schedule of events	Baseline	Procedure	Discharge	30 days + 7 days	2 years ± 30 days	5 years ± 30 days	Unscheduled visit
**Informed consent**	X						
**Demographics and medical history**	X						
**Risk analysis for hernia**	X						
**QOL (RAND-36, AAS, PROMIS)**			X	X	X	X	
**Procedure details**		X					
**Clinical evaluation**	X		X	X	X	X	X
**Ultrasound findings**					X	(X*)	(X)
**Protocol deviation**	X*	X*	X*	X*	X*	X*	X*
**Complications**		X*	X*	X*	X*	X*	X*
**Study closure form**						X†	

* Complete if applicable. †Complete when lost to follow-up, if there is consent withdrawal, or the subject completed all study-related visits. QOL, quality of life; AAS, Activities Assessment Scale.

Basline data include: age; BMI; Charlson Co-morbidity Index; previous abdominal surgical history; history of smoking; previous hernias; previous hernia-related operations; WHO class of physical activity; medications affecting healing (corticosteroids, immunosuppressive medications, biologicals); creatinine; international normalized ratio (INR); albumin; informed consent and patient information; and randomization group.

Intervention data include: prophylactic antibiotics given; ASA physical status class; presence of hernias in midline; presence and width of rectus diastasis; contamination class (I clean, II clean contaminated, III contaminated, IV dirty, infected); surgical procedure; International Statistical Classification of Diseases and Related Health Problems diagnostic code (ICD-10); blood loss; time taken to create the retrorectus space and insert the mesh; length of wound; suture material and needle used; drains left; vacuum-assisted closure/other temporary closure/skin left open; skin closure.

Data on primary hospital stay and discharge include: SSI rate; all complications during hospital stay measured by CCI and Clavien–Dindo classification; reoperations; fascial dehiscence; duration of stay; mesh removal; and place of discharge.

#### Thirty-day follow-up

All patients will be contacted by telephone 30 days after surgery. If there are any deviations from the recovery, the patient will be invited to the outpatient clinic for a follow-up visit. Information gathered at follow-up will include: return to previous level of activity; return to work, length of sick leave; bulging; wound status; complications measured by Clavien–Dindo classification; readmissions; reoperations; removal of mesh; QOL (RAND-36, AAS, PROMIS); and protocol deviations.

#### Two-year follow-up

Patient-related recovery outcomes and QOL questionnaires (RAND-36, AAS, PROMIS) will be completed, and any complications, clinical signs, and abdominal ultrasound findings of an incisional hernia or protocol deviations will be reported. Both the patient and surgeon assessing recovery and well-being of the patient will be blinded to the randomized groups.

The ultrasound findings will all be analysed by a single independent radiologist at each study site who will be blinded to the randomized groups. Possible hernia opening, size, location and incisional sac volume will be defined both at rest and with the Valsalva manoeuvre. If the findings are inconclusive or there is a discrepancy between the clinical assessment and imaging, or a patient has a symptomatic incisional hernia and operative treatment is indicated, an abdominal CT scan will be done to verify the hernia diagnosis or plan an operative technique.

#### Five-year follow-up

Patient-related functional outcomes and QOL will be completed and any complications, clinical signs of an incisional hernia, or protocol deviations will be reported. Additionally, ultrasound scans will be done following the same protocol as described for the 2-year control if there is any suspicion of incisional hernia.

All exceptions to the protocol will be recorded and explained in detail at each point of the follow-up schedule.

### Sample size

To calculate the sample size required to compare these two groups, based on previous studies^12,13^, a 10 per cent rate of incisional hernia in the mesh group and a 25 per cent incisional hernia incidence in the control group upon clinical assessment and ultrasound examination was estimated. Assuming α = 0.05 and a power of 80 per cent, 97 patients would be needed per group. Furthermore, assuming a 2-year dropout rate of 20 per cent, 122 patients per group are needed (244 patients in total). The sample size is calculated only for the primary outcome, and the secondary outcomes will be interpreted for hypothesis-generating only. If the estimated 20 per cent dropout rate is exceeded, the sample size may be recalculated. All analyses will be performed by or under the guidance of a professional statistician and following the CONSORT guidelines^17^.

### Recruitment

All patients who are eligible will be offered enrolment in the study at each study site by the surgeon on call. After receiving the information on the possible advantages and disadvantages of the intervention as well as signing the informed consent form, the subject will be enrolled in the PREEMER trial. Documentation given to participants is provided in Finnish and Swedish and available on request. To control selection bias, a prospective screening log of patients not participating in the study will be maintained during the study period.

### Randomization

Preoperative randomization has been chosen over intraoperative randomization to promote randomization of patients with peritonitis and contaminated surgical sites. A dedicated electronic database and randomization software will be used to host the clinical trial data for this study. A separate randomization list will be created for each participating centre. Patients are randomly assigned (in 1 : 1 ratio) to either an intervention group or control group according to a computer-generated list compiled by a biostatistician otherwise uninvolved in the analyses or clinical care or outcome assessment of the trial patients. The allocation will be stratified according to patient BMI (less than 30 and equal to or greater than 30 kg/m^2^), previous laparotomy history (previous midline laparotomy/no previous midline laparotomy), conversion (yes/no) and age (less than 65 and 65 years or more) and blocked within the strata using random permuted blocks (block sizes 2, 4, 6 and 8).

### Blinding

The surgeon performing the intervention will randomize the patient and will not be blinded to the allocated intervention. Study patients will not be informed of the allocated group and will thus remain blinded of the randomized group during the whole follow-up period. Both the surgeon evaluating the outcome at the ward, and at 30-day, 2-year and 5-year follow-ups, as well as the radiologist, will be blinded to the randomized groups. The surgeon, who carried out the intervention and was not blinded, will not be involved in assessing the outcomes or in the treatment of the patient postoperatively. To maintain the blinding of the treating surgeons, in both groups, the following sentence will be written in the medical records instead of revealing the randomized group: ‘Fascial closure was performed according to randomized group’. After the recruitment has ended, but before analyses of the data, the allocated groups will be given arbitrary names (e.g. A and B) and the analyses for primary and secondary outcomes will be carried out without the knowledge of which group is which. Only after the interpretation of the results will the real names of the allocated group be revealed. Patients’ randomization numbers will be available in the medical records. Envelopes marked with the randomization numbers and containing the allocated group information will be accessible at all times in the case of complications etc. A record of unsuccessful blinding will be maintained and published.

### Statistical methods

Based on previous studies on incisional hernia prevention at emergency laparotomies, a 15 per cent decrease in incisional hernia incidence at 2-year follow-up has been assumed^12,13^. All analyses will be performed primarily according to the modified intention to treat principle, in which all randomized patients are included in the analyses, except for patients who were excluded because of intraoperative exclusion criteria.

The primary endpoint will be the incidence difference of incisional hernias with a 95 per cent confidence interval between the study groups during the 2-year follow-up. The primary endpoint as well as other categorical data will be analysed by χ^2^ test or Fisher’s exact test. Student’s *t*-test or Welch test will be used for the continuous variables; the latter only if the assumption of homogeneous variance does not hold. The hernia incidence will also be analysed using Kaplan–Meier analysis. The primary imaging method in the study is ultrasonography. However, mesh-related symptoms may lead to an increased number of CT scans, i.e. lead to increased number of hernia diagnoses. Therefore, sensitivity analysis will be performed including only patients with hernia diagnosed either clinically or by ultrasound. The linear mixed model or generalized linear mixed model will be used for repeatedly measured data, the former for continuous data and the latter for categorical data. The statistical programs SPSS^®^  version 24.0 (IBM, Armonk, New York, USA) and SAS^®^ version 9.4 (SAS Institute Inc., Cary, North Carolina, USA) will be used for the analyses.

### Interim analyses

As previous research on synthetic mesh utilized as prophylaxis at emergency midline laparotomy is scarce, an analysis of the complications and risks will be done and evaluated for safety reasons after 30 patients have been randomized to each group and reached 30 days’ follow-up. For the same reason, there will be further analysis on the complications of the mesh after 30 patients randomized to each group have reached the 2-year follow-up. The results of interim analyses will be communicated with Oulu University ethics committee. If there are significantly more serious complications in either group compared to the other at the 30-day or 2-year control, the trial will be discontinued.

### Adverse event reporting and harms

An eCRF will be filled for any complication that is Clavien–Dindo IIIb or more serious, including complications requiring treatment under general anaesthesia, life-threatening complications requiring intensive care, or death of the patient. If there are significantly more serious complications in either group compared with the other at the 30-day or 2-year control, the trial will be discontinued.

### Data management and collection

A dedicated electronic database and randomization software will be used to host the clinical trial data for this study. All eCRFs are handled with a special trial ID and date of birth. Access to the database is limited to the main investigators, and all data requested on the eCRFs will be recorded. Any missing data will be explained. The data collection will be the responsibility of the principal investigator at each study site and will be reviewed by the study group. The reasons for withdrawal will be documented carefully. The investigator will attempt to contact the subjects at least three times prior to designating them as lost to follow-up. The investigator will document the date and type of attempted communication. If a subject cannot be reached during the visit window, a missed visit is recorded; after three consecutive missed visits, a subject will be considered lost to follow-up and a study exit form will be completed on the electronic database. Any data on a subject’s participation and procedures prior to withdrawal will be analysed within the research.

The data sets generated and/or analysed during the present study will not be publicly available due to Finnish laws on privacy protection but will be available from the corresponding author upon reasonable request, if allowed by the local permissions and Finnish law.

### Ethics

According to previous research on mesh prevention on emergency laparotomies, study patients are not prone to complications due to intervention^2,12,13^. Access to the final data set will be limited to the investigators of PREEMER study. The Ethical Committee at Oulu University Hospital has approved the PREEMER Trial (Application number 3/2020) and given consent to participate. Written, informed consent will be obtained from all participants. All modifications to protocol will be communicated with the ethics committee as amendments. All data are handled with study ID and without identification number. The access to data is limited to the main investigators and study nurse. All investigators and study nurses have agreed to be bound to professional secrecy by signing a contract with each hospital district.

### Dissemination plan

The protocol of the trial will be published at the beginning of the trial. The results concerning the primary endpoint and results of secondary endpoints within 2-year follow-up will be published in an international peer-reviewed journal once included patients have reached the 2-year follow-up. The results of the 5-year follow-up will be published in an international peer-reviewed journal when all included patients have reached the 5-year follow-up.

## Discussion

The aim of this study is to assess the safety and efficiency of preventive self-gripping mesh in incisional hernia prevention for emergency abdominal midline laparotomies in a randomized, patient- and assessor-blinded, multicentre setting. The mesh-closure group will be compared with a control group without a mesh closed via a standard small-stitch closure with a continuous slowly absorbable monofilament suture. The hypothesis is that a significant number of symptomatic incisional hernias and further operations due to the incisional hernia can be prevented with a prophylactic mesh. QOL will be measured throughout the study in both groups to analyse the effect of the prophylactic mesh.

Prophylactic meshes significantly reduce the incidence of incisional hernia in high-risk patient groups^5,16^. As the risk of incisional hernia after abdominal midline laparotomy increases to above 30 per cent^13^, a significant number of hernias could be prevented using a prophylactic mesh in the emergency setting. Onlay mesh has been associated with an increased risk of seroma^10,16^. As an emergency laparotomy, especially one at a contaminated surgical site, is prone to infections and seromas^13^, a retrorectus position was chosen for this study. This position also enables the skin to be left open in cases of contamination level IV.

The use of synthetic materials in contaminated surgical sites has been increasing. However, there are concerns over its potential for mesh-related complications, such as infection, chronic pain, seromas and bowel fistulas, especially in emergency situations like peritonitis and intestinal obstruction^10^. Therefore, it is crucial to evaluate the potential benefits, hernia risk groups, costs, QOL and long-term results in a randomized setting before adopting preventive mesh placement on a large scale.

If a significant number of incisional hernias can be prevented safely by using a mesh, not only will patients benefit from a better QOL, but major healthcare cost savings can be achieved. Each year, about 1650 patients undergo an operation in Finland for symptomatic incisional hernias. According to a French study, the estimated cost for an incisional hernia surgery is 6450 Euros.^17^ The corresponding costs in Sweden are even higher, reaching 9060 Euros per treatment.^18^ Extrapolating this to Finland, this means that the operative treatment of incisional hernias costs the Finnish healthcare sector more than 10 million Euros annually. A majority of these costs may be avoided by using the prophylactic mesh during the closure of midline emergency laparotomies in patients presenting with incisional hernia risk factors.

In the two previous studies on this topic, the SSI rate in a Swiss study was 60 per cent and only 17 per cent in a Spanish study^12,13^. This reflects differences in their patient selection, as there were only subjects with peritonitis in the first study, while all kinds of emergency laparotomies were included in the latter. Neither of the studies included in the only meta-analysis on the topic were randomized controlled trials^2^. There were also many methodological differences in both the mesh itself and the mesh placement in these two studies. The conclusion of the only systematic review paper published on the topic was that there is a limited amount of data available for assessing the effect and safety of the use of prophylactic mesh in an emergency laparotomy setting^2^. Thus, randomized control trials are required to address this important clinical question. Moreover, the EHS guideline group came to the same conclusion in their recommendation report for preventing incisional hernias^15^.

### Trial status

Ethics Committee approval in Oulu University Hospital was received 25 February 2020 for protocol version 1.0 dated 2 January 2020. The recruitment began in April 2020 and is anticipated to be complete in 2022. The study was registered NCT04311788 (http://www.clinicaltrials.gov) prior to its start.

## Declaration

E.M. reports consultancy fees from Medtronic. V.M. reports reports grants from Academy of Finland, Syöpäsäätiö (Cancer Foundation), Vatsatautien Tutkimussäätiö Foundation, Mary and Georg Ehrnrooth’s Foundation, Helsinki University Hospital research funds, Finnish Gastroenterological Society, and Finska Läkaresällskapet; personal fees from City of Vantaa, and University of Helsinki, non-financial support from Astellas, all outside the submitted work. F.M. reports grants and personal fees (consultancy, speaker, grants) from Medtronic, CMR Surgical, Intuitive Surgical, Dynamesh, Bard Davol, all outside the submitted work. The other authors have no conflicts of interest to declare.

## References

[1] Mingoli A, Puggioni A, Sgarzini G, Luciani G, Corzani F, Ciccarone F et al Incidence of incisional hernia following emergency abdominal surgery. Ital J Gastroenterol Hepatol 1999;31:449–453 10575560

[2] Burns FA, Heywood EG, Challand CP, Lee MJ. Is there a role for prophylactic mesh in abdominal wall closure after emergency laparotomy? A systematic review and meta-analysis. Hernia 2020;24:441–447 31641872 10.1007/s10029-019-02060-1 PMC7210219

[3] Jeppesen M, Tolstrup M-B, Gögenur I. Chronic pain, quality of life, and functional impairment after surgery due to small bowel obstruction. World J Surg 2016;40:2091–2097 27384171 10.1007/s00268-016-3616-9

[4] Thorup T, Tolstrup M-B, Gögenur I. Reduced rate of incisional hernia after standardized fascial closure in emergency laparotomy. Hernia 2019;23:341–346 30684103 10.1007/s10029-019-01893-0

[5] Muysoms F, Antoniou S, Bury K, Campanelli G, Conze J, Cuccurullo D et al European Hernia Society guidelines on the closure of abdominal wall incisions. Hernia 2015;19:1–24 25618025 10.1007/s10029-014-1342-5

[6] Patel SV, Paskar DD, Nelson RL, Vedula SS, Steele SR. Closure methods for laparotomy incisions for preventing incisional hernias and other wound complications. Cochrane Database Syst Rev 2017; (11)CD005661 29099149 10.1002/14651858.CD005661.pub2 PMC6486019

[7] Tolstrup M-B, Thorup T, Gögenur I. Chronic pain, quality of life and functional impairment after emergency laparotomy. World J Surg 2019;43:161–168 30178128 10.1007/s00268-018-4778-4

[8] Jairam AP, Timmermans L, Eker HH, Pierik R, van Klaveren D, Steyerberg EW et al Prevention of incisional hernia with prophylactic onlay and sublay mesh reinforcement versus primary suture only in midline laparotomies (PRIMA): 2-year follow-up of a multicentre, double-blind, randomised controlled trial. Lancet 2017;390:567–576 28641875 10.1016/S0140-6736(17)31332-6

[9] Warren J, Desai SS, Boswell ND, Hancock BH, Abbad H, Ewing JA et al Safety and efficacy of synthetic mesh for ventral hernia repair in a contaminated field. J Am Coll Surg 2020;230:405–413 31954819 10.1016/j.jamcollsurg.2019.12.008

[10] Lima H, Rasslan R, Novo F, Lima T, Damous S, Bernini C et al Prevention of fascial dehiscence with onlay prophylactic mesh in emergency laparotomy: a randomized clinical trial. J Am Coll Surg 2020;230:76–87 31672681 10.1016/j.jamcollsurg.2019.09.010

[11] Köckerling F, Alam N, Antoniou S, Daniels I, Famiglietti F, Fortelny R et al What is the evidence for the use of biologic or biosynthetic meshes in abdominal wall reconstruction? Hernia 2018;22:249–269 29388080 10.1007/s10029-018-1735-y PMC5978919

[12] Argudo N, Pereira JA, Sancho JJ, Membrilla E, Pons MJ, Grande L. Prophylactic synthetic mesh can be safely used to close emergency laparotomies, even in peritonitis. Surgery 2014;156:1238–1244 25017136 10.1016/j.surg.2014.04.035

[13] Kurmann A, Barnetta C, Candinas D, Beldi G. Implantation of prophylactic nonabsorbable intraperitoneal mesh in patients with peritonitis is safe and feasible. World J Surg 2013;37:1656–1660 23568247 10.1007/s00268-013-2019-4

[14] Borab ZM, Shakir S, Lanni MA, Tecce MG, MacDonald J, Hope WW et al Does prophylactic mesh placement in elective, midline laparotomy reduce the incidence of incisional hernia? A systematic review and meta-analysis. Surgery 2017;161:1149–1163 28040255 10.1016/j.surg.2016.09.036

[15] Muysoms FE, Miserez M, Berrevoet F, Campanelli G, Champault GG, Chelala E et al Classification of primary and incisional abdominal wall hernias. Hernia 2009;13:407–414 19495920 10.1007/s10029-009-0518-x PMC2719726

[16] Slankamenac K, Graf R, Barkun J, Puhan MA, Clavien PA. The comprehensive complication index: a novel continuous scale to measure surgical morbidity. Ann Surg 2013;258:1–7 23728278 10.1097/SLA.0b013e318296c732

[17] Moher D, Hopewell S, Schulz KF, Montori V, Gøtzsche PC, Devereaux PJ et al CONSORT 2010 explanation and elaboration: updated guidelines for reporting parallel group randomised trials. Int J Surg 2012;10:28–55 22036893 10.1016/j.ijsu.2011.10.001

[18] Gillion J, Sanders D, Miserez M, Muysoms F. The economic burden of incisional ventral hernia repair: a multicentric cost analysis. Hernia 2016;20:819–830 26932743 10.1007/s10029-016-1480-z

[19] Millbourn D, Wimo A, Israelsson LA. Cost analysis of the use of small stitches when closing midline abdominal incisions. Hernia 2014;18:775–780 23839330 10.1007/s10029-013-1135-2

